# Parkinson's disease under dopamine replacement therapy as a human framework for studying addiction‐relevant mechanisms

**DOI:** 10.1111/pcn.70118

**Published:** 2026-08-11

**Authors:** Vanessa Fleury, Elvira Pirondini, Damien Benis, Tiffany Corbet, Gabriel Thorens, Christian Lüscher, Paul Krack

**Affiliations:** ^1^ Department of Neurology Geneva University Hospital Geneva Switzerland; ^2^ Faculty of Medicine University of Geneva, Rue Michel‐Servet 1 Geneva Switzerland; ^3^ Rehabilitation Neural Engineering Laboratories University of Pittsburgh Pittsburgh Pennsylvania USA; ^4^ Physical Medicine and Rehabilitation University of Pittsburgh Pittsburgh Pennsylvania USA; ^5^ Bioengineering University of Pittsburgh Pittsburgh Pennsylvania USA; ^6^ Neuroscience of Emotion and Affective Lab University of Geneva Geneva Switzerland; ^7^ Addictology Division, Mental Health and Psychiatry Department Geneva University Hospital Geneva Switzerland; ^8^ Department of Basic Neurosciences, Faculty of Medicine University of Geneva Geneva Switzerland; ^9^ Synapsy Center for Mental Health Research University of Geneva Geneva Switzerland; ^10^ Department of Neurology Inselspital, University Hospital Bern, University of Bern Bern Switzerland

**Keywords:** behavior, dopamine, impulse control disorder, Parkinson's disease, substance‐related disorders

## Abstract

Addiction is a chronic relapsing condition characterized by compulsive reward seeking and impaired behavioral control, associated with major individual and societal burden. Mechanistic investigation in humans remains constrained by ethical limitations, uncontrolled exposure histories, and ecological confounds. Parkinson's disease treated with dopamine replacement therapy provides a human framework for investigating addiction‐relevant mechanisms under controlled dopaminergic modulation. We performed a narrative synthesis of clinical, neuropsychological, pharmacological, neuroimaging, and electrophysiological evidence linking impulse control disorders and dopamine dysregulation syndrome in Parkinson's disease to dimensional and circuit‐based models of addiction. We examined phenotypic and neurobiological convergences, experimental opportunities, and translational relevance while outlining its boundary conditions. A subset of Parkinson's disease patients exposed to dopamine replacement treatment develops impulse control disorders and dopamine dysregulation syndrome. Impulse control disorders comprise heterogeneous behavioral phenotypes that share selected dimensions with behavioral addictions, whereas dopamine dysregulation syndrome most closely approximates pharmacological substance use disorders. These phenotypes share selected addiction‐related dimensions, including craving, impaired control, compulsive reward‐seeking, and persistence despite harm, while showing partial clinical and neurobiological convergence with behavioral addictions and substance use disorders. Parkinson's disease patients with neuropsychiatric fluctuations may represent a sensitized vulnerability state in which dopaminergic modulation dynamically influences reward processing, motivation, and compulsive behaviors. Parkinson's disease under dopamine replacement therapy enables within‐subject dopaminergic manipulation, longitudinal observation, prospective assessment of vulnerability traits, multimodal neurobiological investigation, and reduced environmental confounding. It therefore provides a unique human window for investigating addiction‐relevant mechanisms under controlled dopaminergic modulation, complementing animal models while remaining constrained by disease‐specific boundary conditions.

Addiction is a severe, chronic, and relapsing brain disorder characterized by compulsive engagement with rewarding stimuli, diminished behavioral control, and continued pursuit despite adverse consequences.^1^ Although the term addiction is typically used to refer to substance use disorders (SUDs), addiction can also manifest in the absence of exogenous drugs, most clearly illustrated by gambling disorder. Gambling disorder is currently the only formally recognized behavioral addiction in the Diagnostic and Statistical Manual of Mental Disorders‐5 (DSM‐5),^2^ reflecting the robustness of convergent epidemiological, clinical and neurobiological evidence. Nevertheless, researchers have proposed several other behavioral syndromes with addiction‐like characteristics, including compulsive buying, compulsive sexual behavior, binge eating, and hobbyism, as candidate behavioral addictions.^3^, ^4^, ^5^, ^6^, ^7^, ^8^, ^9^, ^10^, ^11^, ^12^, ^13^ Although current diagnostic frameworks integrate these syndromes only partially,^14^ SUDs and addiction‐like behavioral syndromes share several core dimensions, including impaired control, persistence despite adverse consequences, and maladaptive reinforcement.^1^

Despite decades of research, the neurobiology of addiction remains incompletely understood, particularly with respect to the mechanisms underlying compulsive reward seeking and loss of behavioral control.^15^ Although existing animal models and studies of individuals with SUDs have provided major mechanistic insights, important translational limitations remain, particularly for investigating addiction‐related processes prospectively in humans and in the absence of controllable pharmacological exposure.^16^

Here we argue that Parkinson's disease (PD) offers a unique and underutilized translational human window into addiction‐relevant mechanisms. PD is a progressive neurodegenerative disorder characterized by degeneration of dopaminergic neurons and widespread involvement of other neurotransmitter systems, leading to both motor and non‐motor dysfunction.^17^ A significant subset of patients develops addiction‐like behaviors under dopamine replacement therapy (DRT). Two syndromes are particularly informative. First, impulse control disorders (ICDs) encompass a heterogeneous group of behaviors including pathological gambling, compulsive shopping, hobbyism, hypersexuality, binge eating and punding. As these syndromes share impaired control and compulsive engagement, they likely share core neurobiological mechanisms. Indeed, PD‐ICDs map closely onto behavioral addictions, capturing compulsive engagement and persistence despite harm in the absence of illicit substances. ICDs are typically induced by dopamine agonists, but can also occur in patients treated with levodopa alone.^18^ Second, dopamine dysregulation syndrome (DDS) is characterized by compulsive overuse of short‐acting dopaminergic medication (e.g., levodopa or apomorphine).^19^ DDS most closely approximates the phenomenology of SUD, expressing several hallmark addiction dimensions including craving, dose escalation, impaired control, withdrawal‐like negative affect, and persistence despite harm.^20^

PD‐ICDs and DDS thus offer a rare opportunity to prospectively examine the emergence of compulsive reward seeking in humans. As a human disease‐based framework, PD enables longitudinal observation and controlled dopaminergic modulation, including within‐subject ON/OFF medication assessments such as levodopa challenge paradigms.^21^ It also permits multimodal integration through neuropsychological profiling, neuroimaging (e.g., functional magnetic resonance imaging (fMRI)), and electrophysiological recordings such as electroencephalography (EEG) and local field potentials (LFP) during deep brain stimulation (DBS). It also allows partial dissociation of trait‐vulnerability (e.g., impulsivity, novelty seeking) from drug‐induced plasticity. Compared with classical SUDs, PD‐related addiction‐like behaviors occur in the absence of illicit drug exposure and with substantially reduced environmental confounding.^22^

This review examines the behavioral, neuropsychological, and neurobiological convergence between PD‐related addiction‐like behaviors (ICDs, DDS) and contemporary addiction frameworks, while delineating their key divergences. We propose that PD under DRT provides a unique human disease‐based framework (“human window”) for investigating addiction‐relevant mechanisms under controlled dopaminergic modulation. This claim is supported by five secondary propositions: (1) DDS provides a clinically observable form of compulsive dopaminergic drug use resembling SUD; (2) ICDs represent dopamine‐triggered behavioral phenotypes sharing key features with behavioral addictions; (3) neuropsychiatric fluctuations, characterized by alternating hyper‐ and hypodopaminergic states,^23^ may constitute an early clinically observable state in which dopaminergic modulation dynamically influences reward processing, motivation and compulsive behaviors; (4) the controlled and quantifiable nature of DRT enables longitudinal investigation of addiction‐relevant mechanisms in humans; and (5) this framework captures selected dimensions of addiction biology without reproducing the full developmental and social context of addictive disorders.

Unlike most previous reviews, which have primarily addressed the epidemiology, risk factors, clinical management or neurobiology of ICDs and DDS, this review uses these syndromes within a bidirectional translational framework linking movement disorders and addiction neuroscience. We compare convergences and divergences between ICDs, DDS, and behavioral addictions and SUD, and explore how mechanistic insights from PD may inform addiction neuroscience, while concepts from addiction psychiatry may refine the interpretation of compulsive behaviors in PD.

## Literature Search Approach

This narrative review was developed through iterative PubMed searches conducted during both the preparation of the underlying research project and the subsequent development of the manuscript. Searches focused on addiction neurobiology and experimental addiction models, PD‐related ICDs and DDS, DRT, reward processing, neuroimaging including positron emission tomography (PET), dopamine transporter (DAT) imaging and fMRI, electrophysiology including EEG and LFP, and neuropsychiatric fluctuations. We identified additional references through citation tracking of relevant articles and reviews. We selected references according to their relevance to the conceptual framework of the review. Because we used a narrative rather than systematic approach, the literature selection process may be subject to selection bias. Nevertheless, we sought to mitigate this limitation by covering complementary clinical, neuropsychological, pharmacological, neuroimaging, and electrophysiological domains relevant to the proposed conceptual framework.

## Current Neurobiological Models of Addiction

### From reward to compulsivity: The neurocircuitry of addiction

Insights into the neurocircuitry of addiction have emerged primarily from preclinical animal models, including both rodents and nonhuman primates, complemented by human neuroimaging studies. The mesolimbic dopamine pathway, consisting of projections from the ventral tegmental area (VTA) to the nucleus accumbens (NAc), plays a central role in reward‐related learning, motivational processing and the modulation of cortico‐striatal circuits that support adaptive decision‐making. Seminal work in nonhuman primates using single‐unit recordings demonstrated that phasic activity of mesolimbic dopamine neurons encodes reward prediction error, a computational signal critical for reinforcement learning.^24^ Rodent models employing behavioral paradigms such as conditioned place preference, operant self‐administration, and Pavlovian‐to‐instrumental transfer have shown that the mesolimbic dopamine assigns motivational salience to reward‐predicting cues and facilitates adaptive decision‐making in uncertain or changing environments.^25^, ^26^ More recent work suggests that dopaminergic signaling may be spatiotemporally heterogeneous and context‐dependent,^27^, ^28^ supporting a nuanced view of dopamine as a regulator of learning, motivation, salience, and eventually behavioral adaptation. Human fMRI studies have translated these principles by demonstrating that blood oxygenation level dependent (BOLD) signals in the NAc and VTA correlate with both expected reward value and prediction error during decision‐making tasks involving monetary or drug‐related incentives.^29^, ^30^, ^31^ These mechanisms, which normally support flexible learning and behavioral adaptation, are thought to become progressively dysregulated during addiction, contributing to aberrant salience attribution, persistent reward seeking despite adverse consequences, and loss of behavioral control.

At the microcircuit level, medium spiny neurons (MSNs) within the NAc receive convergent glutamatergic inputs from prefrontal cortical regions, including the ventromedial prefrontal (vmPFC), rostral anterior cingulate (ACC) and orbitofrontal (OFC) cortices, as well as the basolateral amygdala and ventral hippocampus. Dopaminergic afferents from the VTA dynamically modulate the efficacy and plasticity of these excitatory inputs. This tripartite synapse integrates reward value, contextual information, and executive control. Rodent electrophysiological recordings have further shown that phasic dopamine bursts encode the presentation of reward‐predictive cues, while tonic firing modulates baseline motivational drive.^32^ Recent studies indicate that D1‐ and D2‐expressing MSNs in the ventral striatum exhibit partially overlapping and context‐dependent functions, rather than strictly opposing roles, underscoring the distributed nature of reinforcement processing.^28^ Optogenetic studies further demonstrated that phasic activation of VTA dopaminergic neurons is sufficient to reinforce operant behavior and, when repeated, to induce several hallmark features of addiction.^33^, ^34^, ^35^ Local GABAergic neurons and other neuronal populations within the VTA bidirectionally regulate dopamine neuron activity, thereby shaping reinforcement, reward consumption, and aversive learning.^36^, ^37^, ^38^ Together, these findings support a distributed circuit in which dopaminergic, glutamatergic, and GABAergic interactions regulate reinforcement learning and motivated behavior.

Collectively, these findings support the view that addiction arises from progressive dysregulation of neural circuits underlying reinforcement learning and behavioral control. Repeated exposure to addictive substances and rewarding behaviors hijacks the mesolimbic dopamine system, producing strong dopamine phasic responses to reward‐predictive cues and modulating top‐down executive control.^39^, ^40^, ^41^ With repeated drug exposure, drug‐induced synaptic plasticity progressively recruits dorsal striatal circuits in addition to ventral striatal networks. This progression reduces behavioral flexibility and strengthens stimulus–response associations. Compulsivity emerges when reward‐seeking behaviors become increasingly insensitive to outcome devaluation, adverse consequences, and changing goals. Experimental studies indicate that this transition involves drug‐induced plasticity within cortico‐striatal circuits, particularly OFC projections to the dorsal striatum, which contribute to the persistent selection of reward‐seeking actions despite negative outcomes. Although this process has been interpreted as reflecting a shift from goal‐directed to habitual control, increasing evidence suggests that compulsion may also arise from excessive goal‐directed behavior. These mechanisms provide a neurobiological framework explaining how initially adaptive reward‐seeking behaviors evolve into persistent compulsive engagement.^15^, ^42^, ^43^

Human neuroimaging studies extend these findings by showing that addiction is not confined to mesolimbic reward circuitry but involves widespread dysregulation across executive, salience, affective, default mode network (DMN), and memory‐related networks. fMRI and PET studies have shown hypoactivation in the dorsolateral and ventromedial PFC, alongside abnormal signaling in the ACC, insula, hippocampus and amygdala.^41^, ^44^, ^45^, ^46^, ^47^ These abnormalities correlate with impaired inhibitory control, heightened cue‐reactivity, poor decision‐making under uncertainty, and emotional dysregulation.^44^ Alterations in the DMN, a key hub for self‐referential thinking and internal monitoring, have also been reported, contributing to impaired self‐awareness and insight.^47^ Together, these findings support a transition from flexible, goal‐directed control toward habitual and compulsive responding mediated by striatal and prefrontal network reorganization.^45^, ^48^

### Limitations of current experimental addiction models

Despite extensive advances in our understanding of addiction neurobiology, current experimental models, whether preclinical or clinical, face key limitations in capturing the multidimensional nature of addictive behaviors. These limitations impair translational potential and reduce ecological validity, particularly with respect to subjective experience, social context, and individual variability, and the ability to prospectively investigate the emergence of compulsive behaviors under controlled conditions. They also complicate efforts to disentangle preexisting vulnerability traits from neuroadaptations induced by addictive substances or behaviors.

Animal models have been foundational for uncovering the roles of dopamine signaling, reinforcement learning, and neuroplasticity in addiction. However, these models conceptualize addiction primarily as a pharmacological process and are inherently limited in representing subjective states such as craving, loss of control, or distress.^49^ Furthermore, they lack the capacity to simulate human social environments, cognitive complexity, or comorbid psychiatric conditions, all central to real‐world addiction.^50^ Rodent studies also suffer from biological homogeneity and persistent sex bias. Most experiments use genetically similar male animals. Sex differences in hormonal regulation, stress reactivity, and reward processing are well established, yet rarely incorporated into standard experimental frameworks.^51^ Nonhuman primates offer greater anatomical and behavioral proximity to humans, but their use is restricted by ethical concerns, high costs, and small sample sizes.^52^

Human studies, while essential for validating preclinical theories, also face substantial limitations. Ethical constraints preclude experimental drug exposure, and most research focuses on treatment‐seeking or abstinent populations, limiting insight into early vulnerability and relapse dynamics.^53^ Cognitive tasks like delay discounting or cue‐reactivity provide mechanistic clues but fail to reproduce the full complexity of addictive decision‐making.^54^, ^55^ Moreover, individuals with psychiatric comorbidities, who constitute a majority of those with SUDs, are frequently excluded, reducing both generalizability and clinical utility.^56^ Sex and gender remain critically under‐addressed. Women are underrepresented in clinical trials and receive suboptimal care, despite known biological and sociocultural influences on addiction trajectories, and sex‐dependent differences in striatal dopamine regulation.^57^, ^58^, ^59^ Importantly, the PD‐DRT framework may also provide a useful platform for addressing this gap. Although PD is more prevalent in men,^60^ women remain substantially represented in clinical cohorts and exhibit distinct manifestations of ICDs, with compulsive buying and binge eating occurring more frequently in women, whereas hypersexuality and gambling are more common in men.^18^ Because dopaminergic exposure can be quantified and manipulated through medication challenges and longitudinal follow‐up, PD may help disentangle sex‐specific dopaminergic responsivity, behavioral expression, and sociocultural influences.

Finally, the majority of animal and human models adopt a linear, substance‐centric view of addiction, overlooking the fact that addiction‐like behaviors can arise without drug exposure. Epidemiological studies suggest that a substantial proportion of the general population may experience at least one behavioral addiction, often exceeding the global prevalence of SUD, estimated at 2–5%.^61^, ^62^ These gaps underscore the need for integrative human frameworks capable of probing compulsive reward seeking beyond substance exposure.

### The opportunity of PD

Given the limitations of existing animal and human models, PD treated with DRT offers a rare translational window to study addiction‐like behaviors in humans. It allows observation of the emergence of compulsive behaviors in the absence of substance abuse, often without prior psychiatric history, within a medically controlled setting, enabling clearer attribution of causality. As such, PD under DRT provides a human disease‐based framework rather than a conventional experimental disease model, bridging mechanistic insight and clinical relevance. Its conceptual positioning as a translational human framework bridging substance‐related and behavioral addictions, while preserving PD‐specific boundary conditions, is illustrated in Fig. 1.

**Fig. 1:**
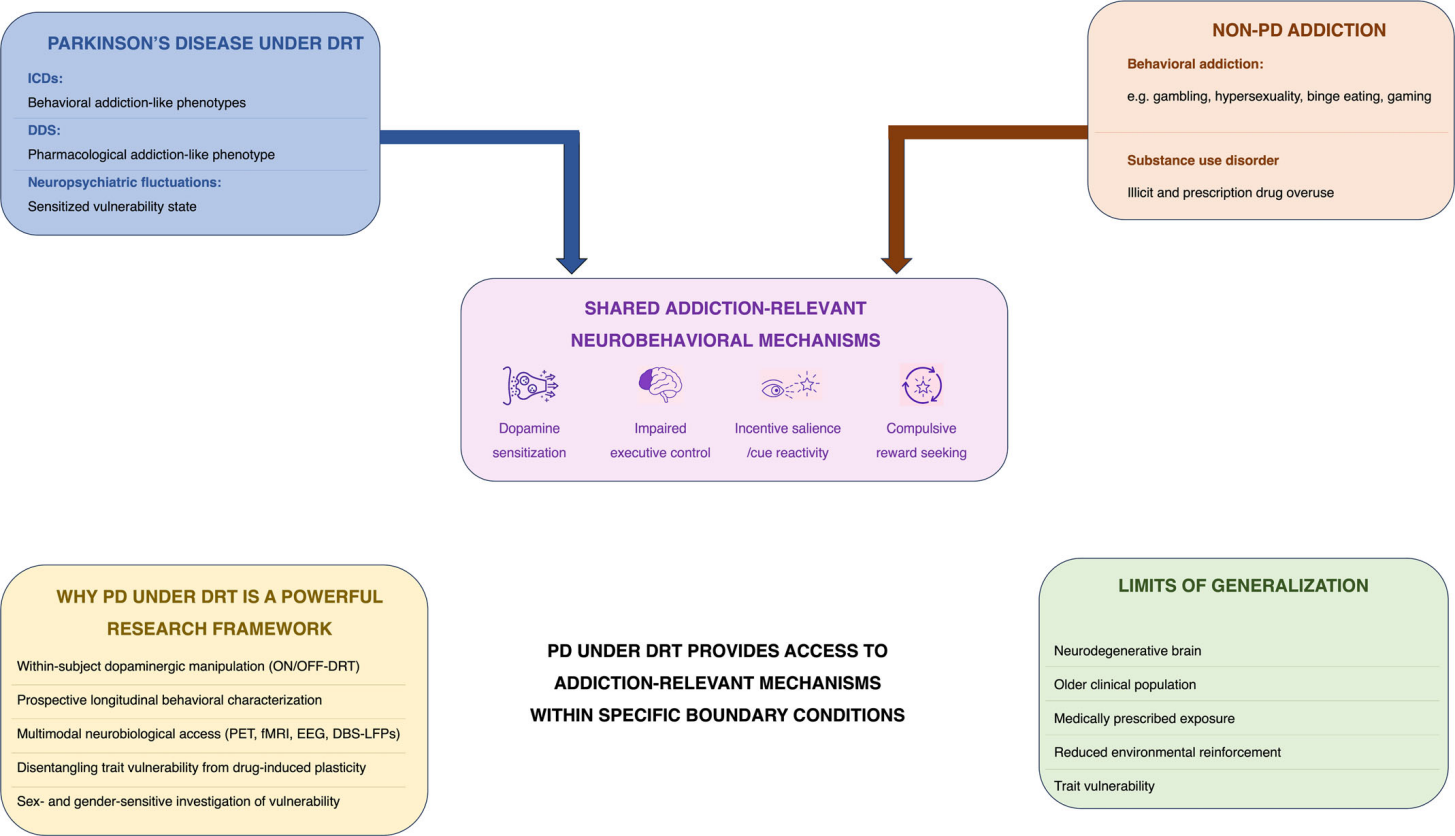
PD under dopaminergic treatment: shared mechanisms and boundary conditions. PD treated with DRT provides a unique human disease‐based framework for studying selected neurobehavioral mechanisms implicated in addictive behaviors. Clinical phenotypes observed in PD under DRT, including ICDs, DDS, and neuropsychiatric fluctuations, provide access to mechanisms that overlap with those involved in behavioral and substance addictions. At the same time, the framework offers unique experimental advantages while remaining constrained by specific disease‐related boundary conditions that limit direct generalization to non‐PD addictions.

## Parkinson's Disease under DRT as a Human Window into Substance‐Related and Behavioral Dimensions of Addiction

PD treated with DRT provides a clinically tractable human disease‐based framework to investigate compulsive reward seeking under controlled dopaminergic modulation. Rather than reproducing addiction as a disease entity, this framework captures specific addiction‐relevant phenotypes, most notably DDS and ICDs within a medically monitored setting. Its advantages are summarized in Table 1. Throughout this review, the term ‘addiction’ refers to the broader neurobehavioral processes underlying non‐PD SUD and behavioral addiction. By contrast, PD‐related ICDs and DDS are considered addiction‐like phenotypes that share selected clinical and neurobiological dimensions with addiction while remaining distinct clinical entities.

**Table 1:** Methodological and translational strengths of PD under DRT as a human disease‐based framework to study addiction

Dimension	Strength within PD under DRT	Boundary conditions
Phenotypic expression	ICDs and DDS express core addiction‐related dimensions (compulsivity, craving, impaired control, persistence despite harm)	Pharmacologically triggered Absence of voluntary drug initiation
Dopaminergic mechanisms	Direct modulation of mesolimbic circuits Convergent PET/fMRI evidence of ventral striatal involvement	Occurs within a neurodegenerative context Not equivalent to SUD and behavioral addictions
Experimental leverage	Within‐subject ON/OFF‐DRT comparisons DBS‐LFP access Longitudinal monitoring	Motor confounds possible
Trait *vs*. State dissociation	Prospective assessment of vulnerability traits before and after DRT exposure	Traits may evolve with disease progression
Clinical accessibility	Stable follow‐up within healthcare systems Structured medication control	Limited generalizability to socially marginalized addiction populations
Ethical feasibility	Dopaminergic titration Levodopa challenge paradigms ethically permissible	Cannot replicate voluntary recreational drug exposure
Translational position	Bridges controlled pharmacology Human compulsive behavior	Captures selected addiction components rather than full ecological trajectory

### Clinical rationale: Converging phenotypes

A subset of PD patients exposed to DRT develop ICDs and DDS.^6^, ^8^, ^9^, ^18^, ^63^ These phenotypes share key clinical features observed across behavioral addictions and SUDs including compulsive reward seeking, craving, impaired control, and persistence despite negative consequences. Neuropsychologically, both SUD and PD patients with ICDs show impaired decision‐making with premature responses and more irrational choices, suggesting higher impulsivity and poor prediction of future rewards compared with PD patients without ICDs and healthy controls.^64^ Phenotypically, DDS most closely approximates a substance‐based addiction‐like phenotype characterized by compulsive dopaminergic medication use, dose escalation and withdrawal‐like dysphoria, whereas ICDs primarily reflect dopamine‐triggered behavioral addiction phenotypes such as pathological gambling, compulsive buying, hypersexuality, or binge eating. PD‐ICDs can nevertheless inform SUD‐relevant mechanisms because they are triggered by dopaminergic therapy and share core neuropsychological dimensions with addictions, supporting a stratified interpretation of addiction‐like features under dopaminergic exposure when key boundary conditions are acknowledged.

Importantly ICDs should not be viewed as a homogeneous entity. Pathological gambling, compulsive buying, hypersexuality, binge eating and punding differ in their behavioral expression, cognitive correlates, and potentially underlying neural mechanisms. Pathological gambling has been most extensively studied and shows the strongest phenomenological overlap with recognized behavioral addictions, whereas other ICD phenotypes may involve varying contributions of impulsivity, compulsivity, reward sensitivity and habit formation.^65^

Consequently, researchers should interpret similarities between PD‐ICDs and behavioral addiction in patients without PD at the level of shared dimensions rather than assuming complete equivalence across all ICD subtypes. This heterogeneity has important methodological implications. Studies should therefore stratify participants by ICD subtype whenever feasible.

### Neurobiological convergence

#### Shared dopaminergic pathways dysregulation

Converging, albeit uneven, evidence implicates mesolimbic dopaminergic dysfunction in SUD, behavioral addictions and addiction‐like behaviors in PD. The strongest evidence concerns the role of the VTA‐NAc dopamine pathways in reward learning, incentive salience, and compulsive reward seeking. In PD, PET studies using [11C]raclopride have reported increased dopamine release in the NAc following levodopa administration in patients with DDS and following reward‐related cues in selected ICD subtypes, particularly pathological gambling.^66^, ^67^, ^68^, ^69^ These findings are broadly consistent with models proposing that repeated dopaminergic stimulation induces progressive behavioral sensitization, and with imaging studies demonstrating increased dopamine release following amphetamine challenge in individuals with SUDs.^70^ However, the available PET evidence remains based on relatively small cohorts, and whether repeated drug exposure produces progressively greater dopaminergic responses remains debated. Rapid dopamine fluctuations occur preferentially within ventral striatal subregions, including the NAc,^71^ and correlate more closely with incentive “wanting” than hedonic “liking”, consistent with incentive sensitization theory.^72^ These signals are thought to contribute to the motivational drive underlying repeated reward seeking and the progressive reinforcement of addictive behaviors.^73^

Several DAT‐SPECT and DAT‐PET studies have reported reduced ventral striatal DAT availability in PD patients with ICDs, particularly pathological gambling.^74^, ^75^, ^76^, ^77^ Similar alterations in DAT availability have also been described in SUD and to a lesser extent, in behavioral addictions, using PET imaging, postmortem studies and preclinical models.^78^, ^79^, ^80^, ^81^ Together, these observations suggest partially shared alterations of mesolimbic dopaminergic function across these conditions.

By contrast, evidence regarding postsynaptic dopamine D2/D3 receptor availability remains more exploratory. PET studies have reported reduced apparent D2/D3 receptor binding availability in the ventral and dorsal striatum of both PD patients with ICDs and patients with SUD. A D3‐selective radioligand PET study found a 20% reduction in apparent D3 receptor binding availability in PD patients with ICDs compared to patients without ICDs, correlating with ICD severity.^82^, ^83^ However, reduced PET binding should not be interpreted unequivocally as reduced receptor density, as it may also reflect increased competition from endogenous dopamine. These findings are conceptually consistent with persistent reductions in striatal D2/D3 receptor binding reported in SUD even months after withdrawal.^84^

Beyond molecular imaging, human fMRI studies support a medication‐dependent amplification of reward sensitivity and value signaling in PD patients with ICDs during reinforcement learning, consistent with a “behavioral addiction‐like” phenotype under dopaminergic stimulation.^85^ This provides an important circuit‐level extension of the molecular imaging literature but should be interpreted as an exploratory observation pending replication.

Overall, PET receptor‐binding and fMRI studies support involvement of mesolimbic dopamine pathways in reward learning, incentive salience and compulsive reward seeking, although evidence beyond dopaminergic dysfunction remains preliminary.

#### Shared large‐scale brain network impairments

Neuroimaging studies have revealed convergent abnormalities within executive, reward, salience, and DMNs in both SUD and PD‐ICDs. Increased connectivity within the reward system, combined with reduced frontostriatal connectivity, has been associated with heightened salience of addictive cues and impaired cognitive control.^86^, ^87^, ^88^, ^89^, ^90^ These abnormalities may precede the development of addiction‐like behaviors, suggesting that network‐level dysfunction or specific neurodegenerative patterns may contribute to vulnerability.^91^

Finally, electrophysiological studies using EEG and LFP recordings further implicate regions involved in motor inhibition, prediction‐error computation, and cue‐related attention in both PD patients with ICDs^92^, ^93^, ^94^, ^95^, ^96^, ^97^ and patients with SUD.^98^, ^99^, ^100^, ^101^, ^102^, ^103^ These abnormalities suggest impaired top‐down control and heightened salience attribution, consistent with clinically observed behavioral dysregulation.

At the circuit level, the overlap between PD‐related addiction‐like behaviors and non‐PD addictions should therefore be viewed as partial rather than complete. Shared abnormalities may involve mesolimbic dopaminergic sensitization, incentive salience, cue‐reactivity, compulsive reward seeking and impaired executive control. However, ICDs and DDS in PD arise within a distinct neurobiological context, in which DRT is superimposed on progressive dopaminergic and non‐dopaminergic neurodegeneration, including serotonergic and noradrenergic systems.^104^, ^105^, ^106^, ^107^, ^108^, ^109^ These disease‐specific conditions may shape reward processing, medication response and compulsive behavior differently from addiction emerging in a structurally non‐degenerative brain.

#### Shared medication/drug mechanisms: DRT as a pharmacological mirror of drugs of abuse

Although administered for therapeutic purposes, DRT shares several pharmacological features with addictive substances. Both ultimately converge on mesolimbic dopaminergic circuits and produce nonphysiological increases in extracellular dopamine, which may promote sensitization and striatal plasticity. As with psychostimulants or opioids,^110^ DRT induces sharp and pulsatile elevations in dopamine levels, particularly when using short‐acting agents such as levodopa or subcutaneous apomorphine.^111^, ^112^ Unlike recreational drug exposure, however, DRT is delivered within a medically supervised setting, providing a unique opportunity to examine addiction‐relevant dopaminergic mechanisms under controlled conditions.

Pharmacologically, DRT includes treatments that either raise extracellular dopamine (e.g., levodopa, COMT inhibitors, MAO‐B inhibitors) or directly activate dopamine receptors (e.g., dopamine agonists). Like addictive drugs, these treatments differentially engage dopamine receptor subtypes.^113^ At a circuit level, both drug classes can shift reinforcement learning toward cue‐driven “wanting” and reduce top‐down control, particularly under intermittent dosing that favors sensitization and habit formation.^114^, ^115^, ^116^, ^117^ Dopamine agonists primarily stimulate D2‐like receptors and provide a relatively continuous pattern of receptor stimulation, whereas levodopa provides broader D1‐ and D2‐receptor activation and can generate marked pulsatile dopaminergic stimulation.^112^ Through combined D1‐ and D2‐receptor engagement and nonphysiological pulsatile kinetics, DRT can promote aberrant cortico‐striatal reinforcement, particularly in sensitized mesolimbic systems. This pharmacological profile is especially relevant for DDS as a proxy of SUD, where repeated dopaminergic “peaks” are associated with escalation and withdrawal‐like negative affect.^118^

In clinical practice, dopamine agonists are associated with a significantly higher prevalence of ICDs than levodopa (14% *vs*. 7.2%), and their combination further increases this risk (17.7%).^18^ This association likely reflects the relatively high affinity of dopamine agonists for D3 receptors, which belong to the D2‐like receptor family and are enriched within mesolimbic regions, particularly the ventral striatum (e.g., islands of Calleja).^119^ Given the central role of these circuits in reward processing, incentive salience, and reinforcement learning, preferential D3 stimulation may provide one of the most direct pharmacological links between dopaminergic therapy and compulsive reward‐seeking behavior, making D3‐mediated signaling a plausible substrate for addiction‐like behaviors under DRT. PET studies using the D3‐preferring radioligand [11C]‐(+)‐PHNO in PD patients with ICDs further implicate this pathway, demonstrating altered D2/D3 receptor availability and associations with ICD severity.^82^, ^83^, ^120^ Beyond PD, [11C]‐(+)‐PHNO PET studies in SUD have also identified abnormalities in D3‐rich regions linked to craving and dopaminergic sensitization, supporting a broader role of D3‐mediated mechanisms in addiction‐relevant motivational processes.^121^ Although these findings remain based on relatively small cohorts and [11C]‐(+)‐PHNO is not exclusively selective for D3 receptors, they support a plausible contribution of D3‐mediated signaling to addiction‐like behaviors.

In contrast, levodopa activates D1‐ and D2‐receptors but can induce more pulsatile dopaminergic signaling. This pulsatility may contribute to sensitization processes and compulsive medication use. Approximately 3–5% of patients develop DDS, which involves compulsive dopaminergic medication overuse, dose escalation, craving, denial, prioritization of medication over social or functional responsibilities, and withdrawal‐like dysphoria during end‐of‐dose periods, resembling SUD despite the absence of illicit drug exposure.^122^ These observations highlight the addiction‐relevant properties of dopaminergic therapy and support its value as a pharmacological analogue of drugs of abuse for investigating compulsive reward seeking within a clinically controlled framework.

### Experimental tractability: Rare human access to mechanisms

PD under DRT enables ethically and methodologically rigorous investigation of addiction‐like behaviors in humans, offering precise dopaminergic manipulation, longitudinal follow‐up, and experimental opportunities rarely attainable in addiction research.

In contrast to addiction research, where repeated experimental induction of withdrawal states is ethically constrained, clinicians can safely achieve dopaminergic modulation in PD patients during the fluctuation stage through ON/OFF‐DRT assessments (i.e., levodopa challenges).^21^ This unique feature allows for within‐subject comparisons of dopaminergic stimulation and withdrawal states, enabling repeated neuropsychological assessments and multimodal imaging (fMRI, PET) across pharmacological conditions.^77^, ^85^, ^93^, ^123^ In select cases undergoing DBS, intracranial electrophysiology provides real‐time access to human reward circuitry.^124^, ^125^

Moreover, PD patients are readily embedded within healthcare systems and regularly monitored, facilitating recruitment, retention, and longitudinal study. This differs from individuals with SUD or behavioral addictions, who often remain outside formal care structures, making systematic longitudinal research more challenging.^126^

### Theoretical advantages: Disentangling vulnerability and drug effects

Addiction is thought to emerge from a complex interplay between pharmacological exposure and predisposing individual traits such as impulsivity, novelty seeking, and altered reward sensitivity. In SUD research, disentangling these components is notoriously difficult. Patients are frequently recruited after years of drug exposure, obscuring the direction of causality between preexisting vulnerabilities and drug‐induced neuroadaptations. Moreover, sociocultural variables, polysubstance use, and uncontrolled drug histories further limit internal validity.^44^ As a result, it remains difficult to determine whether many neurobiological abnormalities observed in addiction represent causes, consequences, or interacting amplifiers of addictive behavior.

In contrast, PD offers a rare naturalistic model to isolate the effects of DRT from preexisting individual differences. Researchers can prospectively assess cognitive and behavioral traits such as delay discounting, reward responsiveness, or risk‐taking before DRT initiation, and track them longitudinally.^74^, ^127^, ^128^ This allows researchers to test how stable, trait‐like vulnerabilities interact with dopaminergic pharmacotherapy to shape the emergence of compulsive behaviors. Empirical evidence supports this view: PD patients who develop ICDs exhibit greater novelty seeking, stronger reward sensitivity, and reduced punishment sensitivity even before ICD onset.^129^ These traits are similarly observed in patients with SUD and their non‐using first‐degree relatives,^130^, ^131^ supporting a contribution of trait‐level vulnerability in addition to pharmacological effects. Genetic studies also suggest shared susceptibilities, with DRD2, DRD3, and GRIN2B polymorphisms linked to both SUD and PD‐ICDs.^132^, ^133^ Together, these findings support a model in which dopaminergic therapy interacts with preexisting behavioral and genetic vulnerabilities rather than acting as a sufficient cause of addiction‐like behaviors.

### Selecting PD subtypes for mechanism‐focused investigation

PD is not a unitary construct. The expression of addiction‐like behaviors varies widely across PD subgroups. To maximize construct validity and specificity, it is essential to align clinical subtypes of PD with the addiction‐relevant processes under investigation. Below, we outline three clinically and mechanistically distinct subgroups that may serve complementary research purposes.

#### PD with DDS: A human analogue of pharmacological addiction‐like processes

DDS is the PD phenotype that most closely resembles pharmacological addiction. Its hallmark is compulsive overuse of DRT, particularly levodopa or subcutaneous apomorphine, despite medical and psychosocial harm.^13^ In early stages, DDS is often accompanied by pronounced neuropsychiatric ON–OFF fluctuations, with euphoric “highs” during ON‐DRT states and dysphoric “lows” during OFF‐DRT states. This profile makes DDS the most direct PD analogue of substance‐related addiction processes within a medically monitored framework.

#### PD with ICDs (excluding DDS): A framework for behavioral addiction mechanisms

ICDs (excluding DDS) are more prevalent (up to 30–40% if hobbyism is included) in PD patients and share core phenomenological dimensions with non‐PD behavioral addictions. They provide the closest PD framework for studying behavioral addiction‐like mechanisms.^9^ Because they are dopamine‐triggered and in the absence of illicit drug exposure, they offer a controlled human context to investigate reward sensitivity, impulsivity, cue‐reactivity, incentive salience and decision‐making under risk. However, given the heterogeneity of ICD phenotypes, future studies should preferentially perform phenotype‐specific analyses and avoid pooling all ICDs into a single category whenever sample size permits. Findings should therefore be interpreted both at the level of specific ICD subtypes and at the level of shared addiction‐relevant dimensions.

#### PD with neuropsychiatric fluctuations: A sensitized, high‐risk state for compulsivity

Patients with neuropsychiatric fluctuations experience marked euphoria during ON‐medication periods and apathy, anxiety, or dysphoria during OFF states.^23^ These fluctuations may mimic the binge/intoxication and withdrawal/negative affect cycles seen in addiction. From an addiction neuroscience perspective, they may reflect an early clinically observable sensitized state in which dopaminergic stimulation acquires enhanced motivational and hedonic significance. Repeated transitions between OFF‐related dopaminergic depletion and ON‐related states of enhanced dopaminergic stimulation may progressively increase mesocorticolimbic responsivity, promoting a sensitized state characterized by fluctuations in mood, motivation, cognition, and reward sensitivity. Consistent with this hypothesis, the only prospective study to date showed that PD patients with neuropsychiatric fluctuations were significantly more likely to develop both ICDs and DDS.^134^ ON‐medication euphoria, rather than OFF‐dysphoria, was most strongly associated with addiction‐like behaviors, suggesting that dopamine‐induced hedonia may act as a gateway to compulsive engagement. This interpretation is consistent with a hypo‐ *versus* hyperdopaminergic behavioral spectrum in PD, in which neuropsychiatric fluctuations may represent a dynamic transition between apathy and reduced reward responsiveness on one side and euphoria, ICDs, and DDS on the other.^135^, ^136^ Unlike SUD, where dopaminergic sensitization is usually inferred only after compulsive behaviors have emerged, PD offers a unique opportunity to prospectively observe the transition toward compulsive reward seeking before overt ICDs or DDS develop. This population may therefore provide a valuable framework for investigating the mechanisms underlying vulnerability to compulsive reward seeking, and the psychostimulant effects of DRT.

#### Should studies select patients with greater or lesser mesolimbic degeneration?

A central question remains: how does the extent of mesolimbic neurodegeneration influence vulnerability to addiction‐like behaviors in PD?

On one hand, the “overdose hypothesis” posits that DRT, titrated to restore dopamine levels in the dopamine‐depleted dorsal striatum, may relatively “overdose” the less affected ventral striatum and PFC, thereby inducing abnormal salience attribution, reinforcement learning, and impulsivity.^137^, ^138^, ^139^ This hypothesis is supported by the occurrence of ICDs in patients treated with dopamine agonists for non‐degenerative conditions (e.g., restless legs syndrome (RLS),^10^, ^11^, ^12^, ^122^, ^140^, ^141^, ^142^, ^143^ prolactinoma,^144^, ^145^, ^146^ fibromyalgia^147^), suggesting that mesolimbic neurodegeneration is not a prerequisite for ICDs.

Nevertheless, direct comparisons suggest that the prevalence of ICDs is significantly higher in PD than in non‐degenerative diseases such as RLS.^7^ This disparity suggests that dopaminergic overstimulation alone cannot fully account for addiction‐like behaviors and that disease‐related changes within mesolimbic networks also contribute to vulnerability. Rather than representing competing explanations, the degree of mesolimbic degeneration and dopaminergic overstimulation likely interact dynamically, with the clinical expression of addiction‐like behaviors depending on both the state of underlying neural circuits and the pattern of dopaminergic exposure.

On the other hand, animal and imaging studies suggest that progressive dopaminergic denervation impairs synaptic dopamine clearance, leading to exaggerated, pulsatile dopamine peaks with each DRT dose,^148^ particularly during the fluctuation stage of PD. Such intermittent dopaminergic stimulation resembles the pattern of repeated drug exposure surges seen in SUD and has been proposed to promote sensitization and compulsive reward seeking. Evidence supporting enhanced mesolimbic dopamine release is strongest for DDS, in which PET studies have demonstrated sensitized ventral striatal dopamine transmission following levodopa administration.^66^ By contrast, PD patients with ICDs show more indirect evidence of mesolimbic dopaminergic dysfunction, including reduced ventral striatal DAT availability and altered dopaminergic PET measures, whereas evidence for enhanced dopamine release remains limited and heterogeneous across ICD subtypes.^75^, ^76^, ^149^ This temporal sequence differs from that observed in SUDs, where mesolimbic dopaminergic abnormalities generally emerge after prolonged drug exposure, making it difficult to distinguish preexisting vulnerability from drug‐induced neuroadaptations.^73^ In PD, by contrast, dopaminergic degeneration precedes the emergence of addiction‐like behaviors, providing a complementary framework to prospectively investigate the interaction between preexisting dopaminergic dysfunction and subsequent drug exposure.

Overall, both relative preservation of mesolimbic circuits (favoring dopaminergic overstimulation) and more advanced mesolimbic degeneration (promoting dopamine sensitization) may confer risk, albeit *via* distinct mechanisms. These are not mutually exclusive and may operate at different stages of disease or interact with individual traits (e.g., reward sensitivity, impulsivity).

#### Implications for study design

These conceptual distinctions translate directly into methodological recommendations, which are summarized in Table 2. Whenever possible, studies should distinguish between individual ICD phenotypes rather than combining them into a single outcome measure, as gambling, hypersexuality, compulsive buying, binge eating and punding likely differ in their underlying mechanisms.^150^, ^151^

**Table 2:** Key design recommendations when using PD under DRT as a human window to investigate addiction

Design strategy	Objectives	Limitations/considerations
Stratify by PD phenotype	Separate PD cohorts with DDS, ICD subtypes, and neuropsychiatric fluctuations to align clinical subtype with addiction‐related profiles (SUD, behavioral addiction, sensitization respectively)	Accurate clinical phenotyping required Subtypes may overlap or evolve over time
Pre‐DRT longitudinal designs	Clarify the temporal sequence of vulnerability and emergence of compulsive behaviors	Requires long‐term follow‐up and high participant retention Disease progression may confound effects
Baseline trait profiling	Assess impulsivity, novelty seeking, delay discounting and reward sensitivity to identify predisposing factors and disentangle vulnerability from drug effects	Traits may be influenced by PD progression or medication Use matched control groups
Age‐ and disease‐matched controls	Distinguish effects of aging and neurodegeneration from addiction‐related mechanisms	Perfect matching is difficult to achieve
ON/OFF‐DRT paradigms	Compare behavioral, cognitive, and neural responses between medicated (ON‐DRT) and unmedicated (OFF‐DRT) states to assess within‐subject dopaminergic modulation	OFF‐DRT state can be uncomfortable in some patients Motor symptoms may affect task performance
Pharmacological stratification	Compare effects of levodopa *vs*. dopamine agonists to explore differential involvement of D1, D2, D3 receptors in compulsive behaviors	Pharmacodynamic differences and receptor selectivity require careful dose matching and timing
Multimodal neuroimaging (fMRI, PET, EEG, LFPs)	Characterize circuit‐level dysfunction	Interpretation should account for disease‐related noise and neurodegeneration‐driven signal changes
Characterization of mesolimbic integrity	Use PET imaging with D2R/D3R or D3R‐selective ligands (e.g., [^11^C]raclopride PET, [^11^C]PHNO PET) to quantify ventral striatal dopamine function	PET access is limited and costly Interpretation requires expertise
Avoid overgeneralization	Focus on specific addiction dimensions (e.g., sensitization, impulsivity, incentive salience)	Does not capture the full ecological trajectory of addiction

## Limitations of PD under DRT as a Human Framework for Studying Compulsive Reward Seeking

While PD under DRT offers a valuable human framework for studying addiction‐relevant mechanisms, it should not be interpreted as a complete model of addiction. The key boundary conditions limiting generalization to non‐PD addictions are discussed below and summarized in Table 1.

### Demographic disparities

Addiction‐like behaviors in PD occur in older patients,^60^ whereas SUD typically emerges in younger individuals.^152^ This age divergence is explained by the age of PD onset but also involves the developmental regulation of traits central to addiction vulnerability, such as impulsivity, novelty seeking, and risk‐taking. Age‐related cognitive changes as well as PD‐related apathy and cognitive decline may also confound assessments of reward sensitivity and compulsivity.

These challenges can be partially addressed through age‐matched control groups, careful cognitive stratification, and prospective designs assessing cognitive and motivational traits before and after DRT initiation.

### Neurobiological disparities

PD‐related addiction‐like behaviors arise in a neurodegenerating brain. PD primarily affects dopaminergic neurons in the nigrostriatal pathways, while mesolimbic dopaminergic pathways are relatively preserved during the early stages of the disease but become increasingly involved as PD progresses. PD also involves non‐dopaminergic systems. As degeneration advances, dopaminergic signaling becomes increasingly dysregulated, and DRT may elicit exaggerated pulsatile dopamine release.^153^, ^154^ Repeated exposure to such pharmacologically imposed dopaminergic surges is thought to induce long‐lasting synaptic plasticity within cortico‐striatal circuits, including potentiation of excitatory afferents onto D1 receptor‐expressing MSNs, a mechanism proposed to contribute to behavioral sensitization and persistent reward seeking despite negative consequences.^16^

By contrast, sensitization in patients with addiction without PD typically develops within structurally intact neural systems through repeated voluntary engagement with addictive substances or behaviors, reinforced by associative learning and exposure to environmental, affective, and contextual cues.^155^ Thus, although PD‐related addiction‐like behaviors and addictions in patients without PD may share dopamine‐dependent modulation of glutamatergic synaptic plasticity, their etiologies, neuroadaptive mechanisms, and behavioral drivers differ.

The present framework adopts a deliberately dopamine‐centered perspective. While justified by the pharmacological nature of DRT, this approach does not fully capture the broader neurobiological, stress‐related, and social and environmental mechanisms that contribute to addiction.

### Environmental and etiological differences

Many addiction models emphasize the role of environmental and psychosocial risk factors, including early trauma, peer influence, and drug availability.^156^ In contrast, PD‐related addiction‐like behaviors occur in a therapeutic setting with prescribed medications. Key components of addiction ecology, including illicit drug access, active drug procurement and social reinforcement, are therefore attenuated or absent.

This reduced environmental complexity strengthens experimental control but limits ecological generalizability. PD under DRT is therefore particularly useful for isolating dopaminergic and neurobehavioral mechanisms, but less suited to modeling the full social and environmental context of addiction.

### Differences in learning, conditioning, and circuit drivers

In SUD, addiction is strongly shaped by learned associations between drug effects and contextual, affective, or environmental cues. These conditioned associations contribute to craving, relapse, and persistent drug seeking.^157^ In PD, compulsive behaviors often arise in a medically prescribed context and may occur with weaker external cue conditioning, particularly in DDS, where compulsive levodopa use may escalate in the absence of illicit drug cues or active drug procurement.

Although cue‐reactivity^67^ and habit learning may still contribute to PD‐related compulsive behaviors, especially in ICDs such as gambling, compulsive buying, hypersexuality, or binge eating, the learning environment differs substantially from that observed in SUD. Consequently, PD under DRT cannot fully reproduce the acquisition, maintenance, and relapse processes that characterize addictions driven by repeated exposure to drugs, environmental cues, and socio‐contextual reinforcement.^67^

### Incomplete representation of the addiction spectrum

Despite the similarities discussed above, PD under DRT does not encompass the full clinical, developmental, and societal spectrum of addiction. Several important dimensions are underrepresented or absent, including polysubstance use, drug‐induced toxicity, legal consequences, social marginalization, and prolonged trajectories of illicit substance use. In addition, addiction in the general population is often shaped by complex interactions between developmental factors, social context, environmental exposures, and drug availability, which are only partially represented in PD cohorts.

Consequently, findings derived from PD should not be generalized to all forms of addiction. Rather, this framework is best suited to investigating specific addiction‐relevant mechanisms under controlled clinical conditions. Direct comparisons with non‐PD addiction cohorts remain essential for determining the extent to which observed mechanisms generalize beyond the PD context.

## Discussion

Taken together, the evidence reviewed here indicates that PD under DRT should be considered not as a complete model of addiction, but as a human disease‐based framework for studying dopaminergic sensitization, compulsive reward seeking, and vulnerability to addiction‐like behaviors under controlled dopaminergic modulation. Its main contribution is to place PD‐related ICDs, DDS, and neuropsychiatric fluctuations within a dimensional addiction framework, while explicitly distinguishing shared mechanisms from disease‐specific boundary conditions.

The translational value of this framework is bidirectional. Concepts from addiction neuroscience, including sensitization, incentive salience, craving, and impaired control, can refine the interpretation of compulsive behaviors in PD. Conversely, PD provides a human platform for testing mechanistic hypotheses that are difficult to examine directly in individuals with SUD or other behavioral addictions. This is particularly evident in patients with DDS and neuropsychiatric fluctuations, who may represent a naturally occurring human model of dopaminergic sensitization under controlled pharmacological conditions. Comparative studies between PD‐related phenotypes and non‐PD individuals with SUD or other behavioral addictions may help disentangle dopaminergic mechanisms of compulsivity from mechanisms driven by developmental history, environmental exposure, social context, or illicit drug use.

Future directions should prioritize: (1) standardizing protocols across PD subtypes (e.g., ICDs, DDS, non‐ICD PD patients with neuropsychiatric fluctuations), (2) expansion of multimodal approaches (e.g., PET‐EEG, DBS‐LFPs, digital phenotyping), (3) systematic integration of sex, age, and genetic factors into vulnerability models, and (4) cross‐population comparisons between PD and non‐PD individuals with SUD or other behavioral addiction cohorts to refine transdiagnostic and translational frameworks. Such interdisciplinary efforts may clarify which components of addiction are fundamentally dopaminergic and which depend on broader environmental and developmental contexts.

Beyond its conceptual value, this framework may have tangible translational implications. Studying addiction‐relevant mechanisms in PD under controlled dopaminergic modulation could facilitate the development of objective biomarkers of vulnerability, compulsivity and treatment response using neuroimaging, electrophysiological and digital phenotyping approaches. Such biomarkers may ultimately help identify individuals at risk of ICDs, guide personalized dopaminergic therapies, and inform the development of circuit‐based interventions.

## Conclusion

As addiction neuroscience increasingly adopts dimensional, circuit‐based, and transdiagnostic frameworks, PD under DRT emerges not as a model of addiction *per se*, but as a unique human window into dopaminergic reinforcement and compulsivity. By combining controlled pharmacological modulation with longitudinal clinical observation and multimodal neurobiological access, this disease‐based framework enables mechanistic interrogation of sensitization, value computation, and impaired executive control in humans. Emerging concepts such as neuropsychiatric fluctuations may provide clinically observable markers of mesolimbic sensitization and offer an opportunity to study the transition from fluctuating hypo‐ and hyperdopaminergic states to more persistent addiction‐like behavioral syndromes. Importantly, PD under DRT may represent one of the few human paradigms in which dopaminergic exposure, sensitization, compulsive reward‐seeking vulnerability states, and longitudinal behavioral trajectories can be studied prospectively under controlled conditions.

Positioned at the intersection of neurology and addiction psychiatry, PD under DRT provides a translational platform to delineate shared *versus* disease‐specific mechanisms of compulsivity and potentially inform therapeutic strategies across substance‐related and behavioral addictions.

## Author contributions

V.F., E.P., D.B., T.C., G.T., C.L., and P.K. conceived and designed the study; V.F., E.P., D.B., and T.C. contributed to drafting the text; V.F. prepared the figure. V.F., E.P., D.B., T.C., G.T., C.L., and P.K. critically revised the manuscript, approved the final version, and agree to be accountable for all aspects of the work.

## Funding statement

This study was supported by the Swiss National Science Foundation (Grant No. 310030_170271).

## Disclosure statement

All authors declare that they have no financial disclosures and no conflicts of interest concerning the research related to the manuscript.

## Data Availability

Data sharing is not applicable to this article because no datasets were generated or analyzed during the current study.
